# Mobile Device-Based Struck-By Hazard Recognition in Construction Using a High-Frequency Sound

**DOI:** 10.3390/s22093482

**Published:** 2022-05-03

**Authors:** Jaehoon Lee, Kanghyeok Yang

**Affiliations:** School of Architecture, College of Engineering, Chonnam National University, Gwangju 61186, Korea; jlee1215@jnu.ac.kr

**Keywords:** struck-by accident, high-frequency sound, Doppler effect, Convolutional Neural Network, construction safety

## Abstract

The construction industry experiences the highest rate of casualties from safety-related accidents at construction sites despite continuous social interest in safety management. Accordingly, various studies have been conducted on safety management, wherein recent studies have focused on its integration with Machine Learning (ML). In this study, we proposed a technology for recognizing struck-by hazards between construction equipment and workers, where a Convolutional Neural Network (CNN) and sound recognition were combined to analyze the changes in the Doppler effect caused by the movements of a subject. An experiment was conducted to evaluate the recognition performance in indoor and outdoor environments with respect to movement state, direction, speed, and near-miss situations. The proposed technology was able to classify the movement direction and speed with 84.4–97.4% accuracy and near-misses with 78.9% accuracy. This technology can be implemented using data obtained through the microphone of a smartphone, thus it is highly applicable and is also effective at ensuring that a worker becomes aware of a struck-by hazard near construction equipment. The findings of this study are expected to be applicable for the prevention of struck-by accidents occurring in various forms at construction sites in the vicinity of construction equipment.

## 1. Introduction

According to the International Labour Organization (ILO), even though the proportion of workers in the construction industry only accounts for 7% of workers in all industries in the world, the rate of casualties in the construction industry is approximately 30–40%, which is higher than in other industries [1]. A National Census of Fatal Occupational Injuries conducted by the United States (U.S.) Department of Labor in 2019 reported that the incident rate of fatal occupational injuries in the construction industry has been consistently increasing over the past five years (from 937 cases to 1061 cases) and that the construction industry recorded the highest rate (1061 cases out of 5333 cases, 19.9%) among all industries in 2019 [2]. Furthermore, in a large number of other countries, the highest fatal accident rate was recorded for the construction industry [3]. According to the Occupational Safety and Health Administration (OSHA) analysis report by the U.S. Department of Labor in 2018, over 60% of all fatalities were caused by the fatal four (i.e., falls, electrocutions, struck-by, and caught-in/-between). Statistical data from 2011 to 2015, obtained from the Center for Construction Research and Training (known as, CPWR) Data Center, showed that approximately half of the fatalities caused by struck-by accidents (384 cases out of 804 cases, 47.8%) were related to vehicle collisions and more than half of those accidents (220 cases out of 384 cases, 57.3%) were caused by vehicles at construction sites [4].

In general, construction sites have a complex and dangerous working environment by requiring a large number of workers and by moving construction equipment during work processes. In particular, construction workers located near the equipment are vulnerable to struck-by accidents due to several risk factors (i.e., a decrease in the worker’s risk perception ability due to noise generated during the operation, the blind spot of construction equipment, and a decrease in driver awareness) [5,6]. Construction site monitoring techniques that can reduce the risk of struck-by accident are essential to solving such problems. In the past, a safety monitoring task involved assigning an experienced supervisor at construction sites to monitor workers, which was time-consuming and ineffective [7]. Studies on automated construction site monitoring have been conducted to overcome the limitations of the previous approach [8]. Computer Vision (CV)-based construction site monitoring has been studied for various purposes, from object detection [9,10] to object tracking [11,12] and activity recognition [13,14]. However, this technique has difficulties in collecting visionary data from the construction site due to frequent visual obstructions. Also, to implement computer vision techniques, it is necessary to secure a certain level of illumination [15,16]. Considering that continuous monitoring is important for the prevention of struck-by accidents, the development of a different technique for struck-by accident prevention would be essential for addressing such limitations.

The proximity-based technique has been widely studied in the construction domain to prevent possible collisions at construction sites. This technique utilizes a radio signal which is less vulnerable in data collection compared to the vision-based approach. Radio-frequency identification (RFID), Bluetooth, and magnetic sensing were applied to measure the distance between the equipment and the worker [17,18]. Also, a signal processing method for a Bluetooth-based proximity sensing system was investigated to solve the possible problems (e.g., signal delay, inconsistent performance) that arise when implementing the system at the construction site [19]. Although the proximity-based system has benefits for continuous monitoring, such approaches still require the installation of additional sensors to generate or receive the signal for sensing.

Recently, studies on construction site monitoring based on sound data have been attempted. Compared to CV, the sound is less constrained by the environment (i.e., weather, illumination, obstacles) [15,20] and the data processing weight is relatively light [21]. Also, the sound-based approach doesn’t require the installation of additional sensors considering that the speaker and microphone are generally installed inside the equipment and a mobile device, respectively. Furthermore, sound data can provide crucial movement-related information that is useful for recognizing dangerous situations such as moving direction, speed, and acceleration by learning the patterns of the sound source [15,22]. Sound-based monitoring techniques are studied for productivity monitoring [23,24,25] and safety monitoring [26,27] by applying various Machine Learning (ML) techniques. Most of these previous studies utilized sound data that was generated during a construction operation. Alternatively, some studies in environment sensing utilized a high-frequency sound near the maximum frequency that humans can hear (e.g., 20 kHz) [28,29,30] and extracted information about the surrounding environments. Although the performance of these systems could be influenced by background noise, these studies demonstrated the validity of the system by applying various filtering techniques.

In this study, we proposed a technique to recognize a struck-by hazard between construction equipment and a worker by integrating a high-frequency sound (i.e., 18 kHz) with a Convolutional Neural Network (CNN), a type of ML with proven effectiveness for sound recognition [31] based on the changes in the Doppler effect caused by movements of a subject. To the author’s knowledge, this study is the first attempt that applied a high-frequency sound and mobile device for the prevention of collision accidents in construction. An experiment was conducted to evaluate the recognition performance of construction equipment in indoor and outdoor environments with respect to movement state, direction, speed, and near-miss (i.e., struck-by) situations. The proposed technology was able to classify the movement direction and speeds with 84.4–97.4% accuracy and near-miss situations with 78.9% accuracy. In the research method section of this paper, the characteristics of the developed technology and the experimental environment are explained; in the preliminary data analysis section, basic data analysis and results are explained. Lastly, the results of the recognition using CNN as well as the limitations and future directions of the research are discussed.

## 2. Research Method

### 2.1. Research Framework and Data Processing

**Figure 1:**
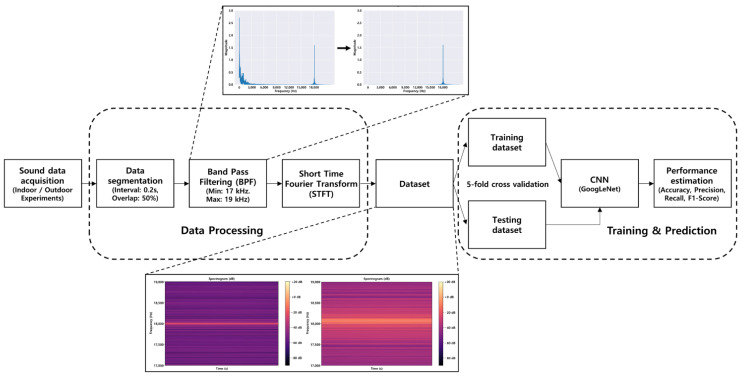
Flowchart of Proposed Movement Monitoring System.

We developed a struck-by hazard recognition technology operable on smartphones, for preventing struck-by accidents occurring between construction equipment and workers. This technology acquired acoustic information having a Doppler effect using the microphone in smartphones, which was then analyzed using CNN, for automatically recognizing the movements in construction equipment and struck-by hazards (see Figure 1). This study utilized the sound of an 18 kHz frequency which is almost inaudible to humans over 18 years of age [32,33]. In detail, the frequency of the sound source used in this study was above the maximum audible frequency of workers in their 20 s or above (17 kHz) [34] and below the sound pickup frequency of a smartphone (20 kHz), which was set to a range where the sound was obtainable even if a Doppler shift occurred. A speaker playing a sound source of 18 kHz was installed on the construction equipment. The sound was then recorded and analyzed using smartphones to recognize dangerous situations. Since most construction equipment is equipped with a speakerphone for alerting movement directions to nearby workers (that is, a backup alarm), we used the device to play the sound source. This technology converted the acoustic information obtained through the microphone on a smartphone into a spectrogram through Band Pass Filtering (BPF) and a Short-Time Fourier Transform (STFT). Figure 1 shows the detailed process of the proposed movement monitoring system.

The sound data obtained in a time-series format through the experiment were segmented into intervals of 0.2 s; 0.5 s of data in the beginning and end were excluded to rule out situations where the construction equipment accelerated, decelerated, or was immobile since the target of the research is recognizing moving directions and speed rather than classifying acceleration and deceleration.

The segmented data were overlapped by 50% with the preceding data for the sake of continuity, and BPF and STFT were implemented to convert it into a spectrogram using the scipy and librosa packages of Python. BPF was used to remove noise other than 18 kHz so that only the data in the range of 17–19 kHz corresponding to ±1 kHz of 18 kHz was used. Subsequently, STFT was used to convert the data into a spectrogram containing time information and frequency domain information. A spectrogram represents time on the x-axis and frequency on the y-axis. In this study, the Doppler shift could be identified from the movement of the construction equipment, nonetheless, a quantitative analysis was challenging due to the nature of the images. However, some studies on situation recognition have been conducted recently by integrating a spectrogram with CNN, which has demonstrated excellent performance in Acoustic Scene Classification (ASC) [35].

In this study, we used GoogLeNet, a CNN model that is the winner of the ImageNet Large Scale Visual Recognition Competition (ILSVRC) 2014 [36]. This model demonstrated outstanding performance in analyzing image data. The GoogLeNet consists of 22 layers, including 9 inception modules that can significantly increase the number of units at each stage without a soaring computational complexity [37]. The GoogLeNet starts with a convolutional layer consisting of a 7 × 7 filter and a 2 × 2 stride and then passes through an activation layer comprising Rectified Linear Units (ReLU) and a max-pooling layer composed of a 3 × 3 pool and a 2 × 2 stride. For the settings of the learning process, the weight learn rate factor and bias learn rate factor were 10 each, the mini-batch size was 16, the initial learning rate was 0.0003, and the maximum epochs were 100. Five-fold cross-validation was applied for a performance evaluation in this study, wherein the performance was analyzed through an average value of each case similar to previous deep learning research [38,39]. Training and testing of the developed model were conducted using the Deep Learning Toolbox of MATLAB, in a desktop computer equipped with an Intel Core i7-11700KF and 32 GB of RAM.

Accuracy, precision, recall, and F1-score were used as metrics for the performance evaluation of the CNN model and the results were visualized through a confusion matrix. A confusion matrix visually indicates the extent of confusion between each category, which is an effective performance metric that can numerically evaluate as well as identify the error type. It is based on a comparison of the predicted results and the actual data. For the data in the actual “True” category, True Positive (TP) denotes a true predicted result, whereas False Negative (FN) denotes a false predicted result. Furthermore, for the data in the “False” category, False Positive (FP) denotes a positive predicted result, and True Negative (TN) denotes a false predicted result [40].

Accuracy, precision, and recall represent the analysis in the diagonal direction, rows, and columns of a confusion matrix, respectively, where accuracy, which evaluates the percentage of correct predictions among all predicted results, is the simplest and most intuitive evaluation metric that is effective for a balanced data scale of all categories (see Equation (1)). Precision and recall are both metrics that evaluate the percentage of correct predictions, where precision evaluates the percentage of true predicted results (*TP*, *FP*), and recall evaluates the percentage of actual true data (*TP*, *FN*) (see Equations (2) and (3)) [41]. Lastly, the *F1-score* is a performance evaluation metric that does not lean toward either precision or recall and uses the harmonic mean of precision and recall (see Equation (4)) [42].

(1) $$Accuracy=\frac{TP+TN}{TP+TN+FP+FN}$$

(2) $$Precision=\frac{TP}{TP+FP}$$

(3) $$Recall=\frac{TP}{TP+FN}$$

(4) $$F1-Score=\frac{(\beta^{2}+1)*\left(Precision*Recall\right)}{\left(\beta^{2}*Precision\right)+Recall}\;≅\;2*\frac{Precision*Recall}{Precision+Recall}$$

### 2.2. Data Collection Environment

**Figure 2:**
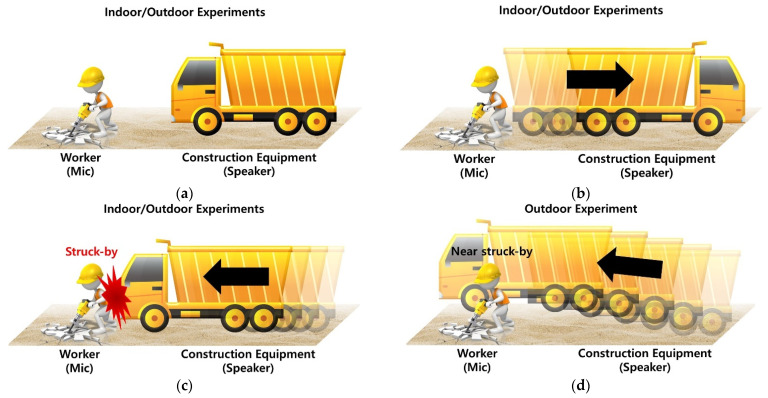
Data Collection Scenarios: (**a**) Stationary Object, (**b**) Moving Backward, (**c**) Moving Forward (struck-by accident), (**d**) Moving Forward (near-miss situation).

An experiment was conducted in both indoor and outdoor environments to evaluate the feasibility of the proposed technology for recognizing a struck-by hazard between construction equipment and a worker. The indoor experiment was conducted to analyze the recognition performance based on the Doppler shift due to the movement of the construction equipment in indoor spaces (that is, moving direction, speeds), whereas the outdoor experiment was conducted to assess the possibility of recognizing the movement of the construction equipment and near-miss (i.e., struck-by) situations in outdoor conditions. Smartphones (iPhone SE 2nd generation, Apple) were used as the experimental devices, considering the universality and future applicability of the technology, along with Bluetooth speakers (Soundcore Motion+, Anker) for generating the almost inaudible sound from the construction equipment. These devices were mounted on a tripod at the height of an average person and on construction equipment to form the experimental environment (see Figure 2 for details). The Ultrasonic Analyzer (an iOS application) was utilized during both experiments with a sampling frequency of 48 kHz for audio data recording. The hearing frequency of the smartphone was examined using an application and it revealed that the embedded microphone supports 20 Hz to 20 kHz sound which is the hearing range of humans. The Bluetooth speaker that was utilized for sound generation in outdoor environments has a 50 Hz to 40 kHz-frequency range and a 30 W audio output.

**Figure 3:**
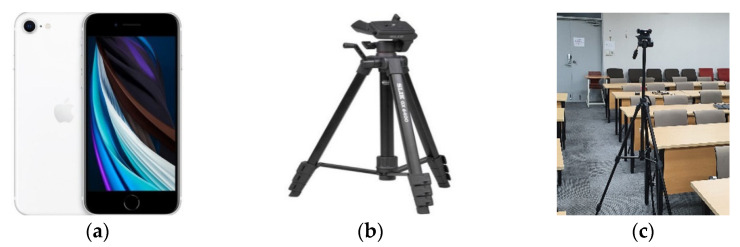
Data Collection Environment (Indoor Experiment): (**a**) Smartphone (iPhone SE2), (**b**) Tripod (ZF-400), (**c**) Experimental Device.

For the indoor experiment, a smartphone was utilized as a sound source instead of construction equipment due to the limited space and possible collision risk. The smartphone was manually moved by a person on a 12 m straight path and an embedded microphone on a smartphone generated the 18 kHz sound during the experiment. (see Figure 3 for details). The indoor experiment was designed considering the most basic form of a struck-by accident or a head-on struck-by between the construction equipment and a worker, wherein the construction equipment moves in a straight line toward the worker. The experiment was conducted according to the following three scenarios: (1) both worker and construction equipment are stationary and maintaining a certain distance (Stationary, ST), (2) worker is stationary and construction equipment is moving away from the worker (Moving Backwards, MB), (3) worker is stationary and construction equipment is moving toward the worker and a struck-by may occur (Moving Forward, MF), as shown in Figure 2a–c. Data were acquired thrice every 25 s for each distance (3 m, 5 m, 7 m, and 12 m) for situation (1), and thrice for when the construction equipment moved at fast (6.2 km/h) and slow (4.32 km/h) speeds on a straight path for situations (2) and (3).

**Figure 4:**
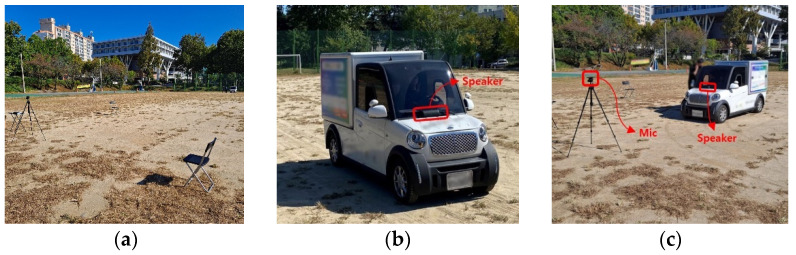
Data Collection Environment (Outdoor Experiment): (**a**) Sensor Setup, (**b**) Moving Vehicle and Speaker Installation, (**c**) Data Collection Process.

A Bluetooth speaker and the embedded microphone in a smartphone were used for the outdoor experiment. Construction equipment was replaced with a vehicle and a worker was replaced with an experimental device on 30 m straight and diagonal paths in an outdoor space resembling a construction site (see Figure 4). The outdoor experiment was designed considering a head-on struck-by between the construction equipment and a worker, as well as a situation where the construction equipment nearly misses colliding with a worker. The following situation was added to the indoor experiment scenarios (1)–(3): (4) construction equipment moves toward the worker but misses (Moving Forward and Near-miss Struck-by, MF_NS), as shown in Figure 2d. Data were acquired thrice every 25 s for each distance (5 m, 10 m, and 15 m) for situation (1), and thrice for when the construction equipment moves at fast (13 km/h) and slow (8 km/h) speeds on straight and diagonal paths for situations (2)–(4).

## 3. Preliminary Data Analysis

**Figure 5:**
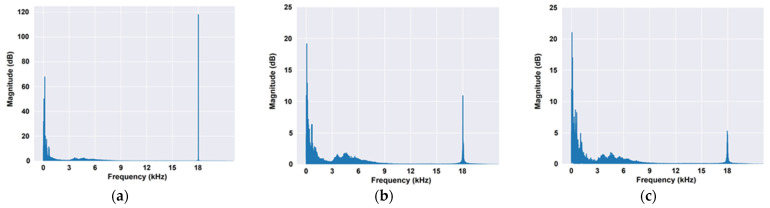
Fourier Transform Results from the Indoor Experiment: (**a**) ST, (**b**) MF_F, (**c**) MB_F.

To evaluate the potential of the movement recognition technology using the Doppler shift, data analysis was first performed using the raw data from the indoor experiment. When the frequency of the data was analyzed using a Fast Fourier Transform (FFT), a range of around 18 kHz, that is, the frequency of the sound source in the experiment, was clearly measured in all experimental data and the Doppler shift was observed (see signal magnitude near 18 kHz in Figure 5) according to the movements between the subjects.

The changes around 18 kHz were further analyzed to examine the changes in the sound data with respect to the Doppler shift (see Table 1). The results showed that the maximum frequency in the situation when there was no movement between the subjects was formed at 18 kHz, the frequency of the sound source. However, the maximum frequency slightly increased to 18.037 kHz when the sound source moved fast toward the worker and slightly decreased to 17.959 kHz when the construction equipment moved fast away from the worker. Therefore, it was verified that the frequency varied according to the movement direction of the construction equipment (that is, moving toward or away from the worker).

**Figure 6:**
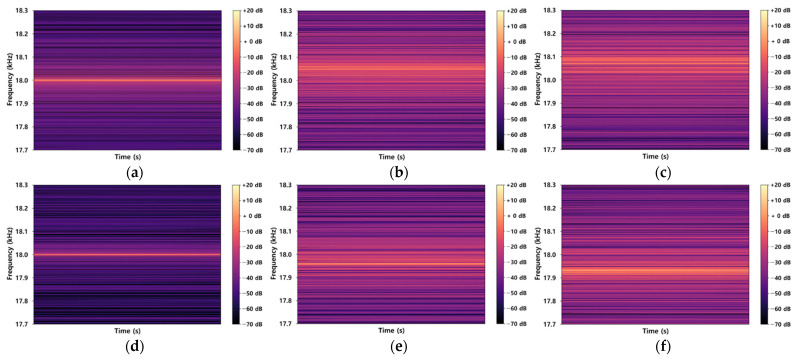
Spectrogram results from the indoor experiment: (**a**) Stationary (3 m), (**b**) Moving Forward (Slow), (**c**) Moving Forward (Fast), (**d**) Stationary (12 m), (**e**) Moving Backward (Slow), and (**f**) Moving Backward (Fast).

Additionally, the data obtained from the indoor experiment were visualized using a spectrogram, as shown in Figure 6, to examine the occurrence of the Doppler shift according to changes in the speeds of the construction equipment. When the range between 17.7 kHz and 18.3 kHz was visualized with respect to the movements of the subjects and the moving speed, 18 kHz was observed when there are no movements, as shown in Figure 6a,d, whereas the Doppler shift was observed when there were movements between the subjects, as shown in Figure 6b,c,e,f. A frequency higher than 18 kHz was observed when the construction equipment moved toward the worker, as shown in Figure 6b,c; however, a frequency lower than 18 kHz was observed when the construction equipment moved away from the worker. A large Doppler shift was visually observed as the moving speed increased when the construction equipment moved toward or away from the worker. This indicated that the moving direction and speed could be analyzed using the sound data obtained from the smartphone. Therefore, in this study, we analyzed the movements of the construction equipment and the struck-by hazard recognition performance of the developed technology.

## 4. Convolutional Neural Network-Based Struck-by Hazard Classification

### 4.1. Struck-by Hazard Recognition—Indoor Environment

In this study, we analyzed the struck-by hazard recognition performance in an indoor environment by dividing the obtained sound data into datasets for recognizing movements (ST, MF, MB), and datasets for recognizing movements and movement speeds (ST, MF_S, MF_F, MB_S, MB_F). The first dataset was configured to analyze the recognition performance when the distance between the construction equipment and the worker was maintained (ST), decreased (MF), or increased (MB). The second dataset was configured to analyze the recognition performance in terms of the movement of the construction equipment (MF, MB) as well as the moving speed (slow, fast). Table 2 lists the amount of data used in each dataset. Data were obtained from various distances (3 m, 5 m, 7 m, 9 m, and 12 m) for ST, thus this class contained more data than the other classes. However, this particular dataset was configured to have the same amount of data as the other classes to eliminate any additional impact.

**Figure 7:**
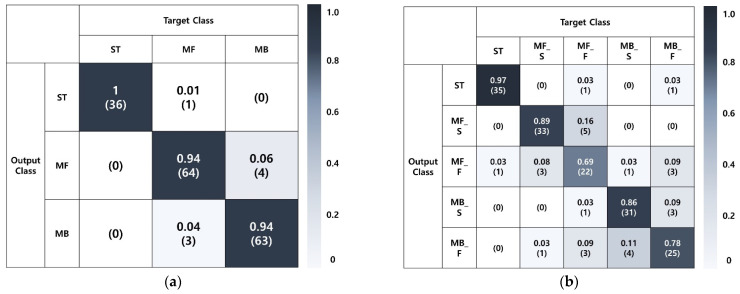
Confusion matrix from the indoor experiment: (**a**) 3 classes, (**b**) 5 classes.

A five-fold cross-validation was used due to the small number of data samples when evaluating the struck-by hazard recognition performance. The validation dataset was not selected in this research due to the small number of data samples. The five-fold cross-validation process divides the entire dataset into five data samples; four data samples (80% of data) were utilized for model training and the remaining data sample (20% of data) was used for model testing. This process was repeated five times until all data samples were applied for testing while changing the configuration of the training sample selection. Figure 7 and Table 3 show the results from the analysis. First, an accuracy of 0.953 was achieved to recognize the movement (3 classes) and high precision, recall, and F1-score (0.94–1) were observed for all classes. An accuracy of 0.844 was achieved to recognize the movement and movement speeds (5 classes). Furthermore, high performance was recorded for ST (precision: 0.946, recall: 0.972, F1-score: 0.959), but lower performance was recorded for the classes including the movement speeds (MF_S, MF_F, MB_S, MB_F) compared with the 3 class datasets (average precision: 0.811, recall: 0.806, F1-score: 0.808). The analysis of the confusion matrix showed that most errors were caused by the confusion of movements in the same direction, which was attributed to a small difference in movement speeds (slow walking, fast walking) in the indoor experimental environment. Considering such errors, the results indicated that the struck-by hazard that could occur indoors was adequately recognized.

### 4.2. Struck-by Hazard Recognition—Outdoor Environment

To evaluate the performance of recognizing struck-by hazards occurring outdoors, the developed technology was evaluated by dividing the data into two datasets in a manner similar to the indoor experiment. The first dataset included five situations for recognizing movements and movement speeds (ST, MF_S, MF_F, MB_S, MB_F), whereas the second dataset included the previous five situations as well as near-miss situations (i.e., struck-by) when the movement speed was slow (MF_NS_S) or fast (MF_NS_F) as the construction equipment moved toward the worker but the struck-by accident did not happen. The struck-by hazard and near-miss situation recognition performance in the outdoor environment was analyzed using the two types of datasets mentioned above; Table 4 lists the total amount of data used in the experiment.

**Figure 8:**
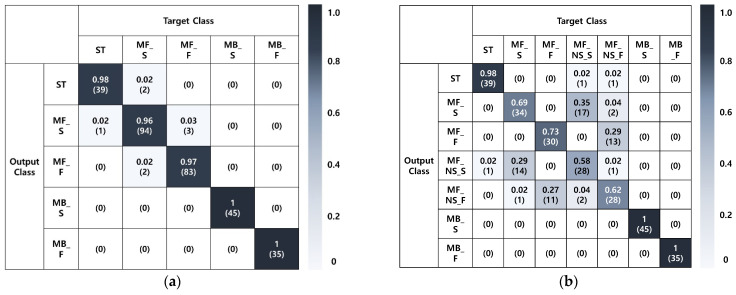
Confusion Matrix from the Outdoor Experiment: (**a**) 5 classes, (**b**) 7 classes.

Furthermore, a five-fold cross-validation was also used to evaluate the struck-by hazard recognition performance in the outdoor experiment and Figure 8 and Table 5 present the performance evaluation results. First, an accuracy of 0.974 was achieved for the 5 classes dataset where precision, recall, and F1-score were all greater than 0.959 for all classes. The analysis of the confusion matrix showed that over half the errors (5 out of 8, 62.5%) were caused by the confusion between MF_S and MF_F. These results implied that the struck-by hazard recognition technology developed in this study was capable of recognizing struck-by hazards in an outdoor environment and could classify the movement speed of the construction equipment to recognize the risk level of hazards. The performance for the seven-class dataset was slightly degraded with an accuracy of 0.789 when compared to the five-class dataset. Specifically, high recognition performance was recorded for non-struck-by hazards such as ST, MB_S, and MB_F (average precision: 0.984, recall: 0.992, F1-score: 0.988) and low recognition performance was recorded for struck-by hazards such as MF_S, MF_F, MF_ NS_S, and MF_NS_F (average precision: 0.661, recall: 0.658, F1-score: 0.659). The analysis of the confusion matrix showed that 98.4% of the total errors (63 cases out of 64 cases, 98.4%) were caused by confusion between the MF classes, particularly, the confusion between a struck-by hazard and a near miss. Specifically, the recognition of a near-miss situation (average precision: 0.652, recall: 0.603, F1-score: 0.627) was more difficult than the recognition of a struck-by hazard. However, most errors found in the dataset were due to misclassification among the struck-by hazard types. Hence, the developed technology was confirmed to be capable of adequately recognizing the struck-by hazards where the distance between the construction equipment and the worker decreased.

## 5. Discussion

Construction work mainly involves manual tasks and has a high risk of safety-related accidents due to the frequently changing working environment. Major accidents at construction sites with high risk and frequency are falls, electrocutions, struck-by, and caught-in/-between. Therefore, in this study, we developed a technology for preventing a struck-by accident between construction equipment and a worker. Since previous struck-by accident prevention technologies focus on recognizing dangerous situations and preventing accidents from the perspective of construction equipment operator, we attempted to overcome the difficulties faced by workers in recognizing a struck-by hazard in real time when construction equipment moves toward them while they are engaged in other tasks near the equipment. In particular, movements of the equipment within a range of possible collisions should alarm nearby workers regardless of an operator’s intention. However, the current approach requires an installation of additional sensors to recognize struck-by hazards.

Therefore, in this study, we developed a technology that can recognize a struck-by hazard when the distance between construction equipment and a worker is decreasing by using data from an embedded microphone in the worker’s mobile device. The developed technology utilized the sound generated by the construction equipment using a Bluetooth speaker and analyzed it to recognize a struck-by hazard. The indoor and outdoor experiments conducted in this study proved that the developed technology could accurately recognize the change in distance between the construction equipment and a worker as well as the change in movement speed. The analysis results showed that the technology demonstrated a high accuracy of 95.3% at recognizing a hazardous situation in an indoor environment with precision, recall, and F1-score values of at least 0.94. The accuracy slightly decreased (that is, 84.4%) when classifying struck-by hazards in terms of movement speed in an indoor environment; however, the performance was adequate since most errors were caused by difficulty in classifying movement speeds.

In an outdoor environment, a high accuracy of 97.4% was demonstrated in struck-by hazard recognition in terms of movement, with precision, recall, and F1-score values of at least 0.951. These results were attributed to the fact that an actual vehicle was used in the outdoor experiment with a greater difference in movement speeds compared to the indoor experiment. Furthermore, a dataset consisting of seven classes was created by classifying the situations where the construction equipment moved toward a worker but did not collide, into different classes to evaluate whether a near-miss situation could be recognized. The analysis results showed that the overall classification performance was considerably reduced to an accuracy of 78.9%, where the F1-scores of a struck-by hazard and a near-miss were between 0.609 and 0.714, thus demonstrating that both situations were difficult to discern. Although the proposed approach is not able to distinguish near-miss situations with over 80% accuracy, the approach has potential uses since it is able to recognize the struck-by hazard with high accuracy (i.e., 97%). Most misclassifications are made between near-miss situations and actual struck-by accident conditions. This fact illustrated that misclassification was observed among the struck-by hazard types, that is, the technology did not classify a dangerous situation as not dangerous or vice versa.

Since the proposed technology did not require additional devices besides a speaker on the construction equipment and a worker’s smartphone, it was highly applicable in construction equipment as well as other moving objects such as hooks on a crane that move vertically. The developed technology was capable of precisely recognizing the movement directions of the construction equipment in indoor environments and the movement directions and speeds in outdoor environments (indoor: 0.953, outdoor: 0.974) using a sound interval of 0.2 s to identify dangerous situations at the construction sites in near real time, thus contributing to the prevention of struck-by accidents and the evaluation of risk levels at construction sites.

The technology developed in this study uses a worker’s smartphone to recognize the movement of nearby construction equipment, thus being capable of accurately recognizing movement directions and speeds; however, it was less effective at recognizing the near-miss situations designed in the experiments. Additionally, the developed technology had the following limitations. First, most construction sites have multiple types of equipment working simultaneously, but this study tested a struck-by hazard situation involving a single type of moving construction equipment. Another limitation was that the struck-by accident that our technology attempted to prevent may occur not only when construction equipment moves toward a worker, but also when construction equipment and a worker move simultaneously. In actual construction sites where different types of machinery are used in a complex manner, it is challenging to recognize a struck-by hazard in real time. Moreover, the proposed technology recognized the movement of a sound source by analyzing the Doppler shift generated from the sound range that is almost inaudible to the human ear; therefore, the movement of the construction equipment may not be recognized if a sound source of the same frequency is generated from multiple types of equipment and becomes overlapped. Accordingly, technology must be developed that will prevent the Doppler shift from the movement of certain equipment from being affected by the sound from other equipment by assigning a unique frequency to each type of construction equipment. As shown in the data analysis results (Table 1), a difference of 40.8 kHz was observed during fast movements in an indoor environment, which indicated that it was possible to set a unique frequency to recognize the movements of diverse construction equipment in a range between the 18 kHz used in the experiment and 20 kHz, which is the sound pickup frequency of a smartphone. Since it is unlikely that multiple types of construction equipment would be used in the same space for the same tasks, setting a specific frequency for each type of construction equipment will be effective for preventing struck-by accidents at construction sites. The developed technology was investigated in both indoor and outdoor environments and the results were analyzed accordingly, but the consideration of the various struck-by hazards and environmental factors such as the vibrations and noise that occurs during construction work, was insufficient. Lastly, the technology must be further developed to exhibit optimal performance by applying various ML algorithms including a Recurrent Neural Network (RNN) capable of handling time-series data and diverse CNN models in addition to GoogLeNet used in this study, for classifying situations.

## 6. Conclusions

Compared to other industries, construction industries across the world experience the highest rate of casualties from safety-related accidents at constructions sites despite continuous social interest in safety management. In this study, we proposed a technology for recognizing a struck-by hazard involving construction equipment, using high-frequency sound and a Convolutional Neural Network, and verified the hazard recognition performance through indoor and outdoor experiments. The proposed technology recognized when the distance between construction equipment and a worker decreased, using the Doppler shift generated from the movement of the construction equipment, which only required a worker’s smartphone, and thus, is highly applicable. Furthermore, the proposed technology could classify the movement direction and speeds of the construction equipment with an accuracy of 84.4–97.4% in indoor and outdoor environments and a near-miss situation with an accuracy of 78.9%. Moreover, the technology developed in this study could prevent struck-by accidents involving construction equipment at construction sites and could contribute to the development of autonomous safety management technology for construction sites. The major limitations of this study were that the technology was evaluated only through experiments rather than at actual construction sites, and that the performance was evaluated for situations where only the construction equipment moved. A follow-up study should be conducted on performance evaluation in actual construction sites with different types of construction equipment and generated noises, as well as the recognition of various struck-by hazards.

## Figures and Tables

**Table 1:** Comparison of Peak Frequencies from the Indoor Experiment.

Categories	Peak Frequency (Hz)	Difference of Peak Frequency from 18 kHz
ST	17,999.7	−0.3
MF_S	18,004.9	+4.9
MF_F	18,037.9	+37.9
MB_S	17,983	−17
MB_F	17,959.2	−40.8

**Table 2:** Datasets from the Indoor Experiment.

Dataset	Categories	Number of Dataset
3 Classes (Indoor)	ST	180
	MF	340
	MB	335
5 Classes (Indoor)	ST	180
	MF_S	182
	MF_F	158
	MB_S	180
	MB_F	155

**Table 3:** Classification performance from the indoor experiment.

Dataset	Categories	Metrics			
		Precision	Recall	F1-Score	Accuracy
3 Classes (Indoor)	ST	0.973	1	0.986	0.953
	MF	0.941	0.941	0.941	
	MB	0.955	0.940	0.947	
5 Classes (Indoor)	ST	0.946	0.972	0.959	0.844
	MF_S	0.868	0.892	0.880	
	MF_F	0.733	0.688	0.710	
	MB_S	0.886	0.861	0.873	
	MB_F	0.758	0.781	0.769	

**Table 4:** Datasets from the Outdoor Experiment.

Dataset	Categories	Number of Dataset
5 Classes (Outdoor)	ST	198
	MF_S	488
	MF_F	434
	MB_S	227
	MB_F	177
7 Classes (Outdoor)	ST	198
	MF_S	244
	MF_F	206
	MF_NS_S	244
	MF_NS_F	228
	MB_S	227
	MB_F	177

**Table 5:** Performance of the Proposed Model in the Outdoor Environment.

Dataset	Categories	Metrics			
		Precision	Recall	F1-Score	Accuracy
5 Classes (Outdoor)	ST	0.951	0.975	0.963	0.974
	MF_S	0.959	0.959	0.959	
	MF_F	0.976	0.965	0.971	
	MB_S	1	1	1	
	MB_F	1	1	1	
7 Classes (Outdoor)	ST	0.951	0.975	0.963	0.789
	MF_S_S	0.642	0.694	0.667	
	MF_S_F	0.698	0.732	0.714	
	MF_NS_S	0.636	0.583	0.609	
	MF_NS_F	0.667	0.622	0.644	
	MB_S	1	1	1	
	MB_F	1	1	1	

## Data Availability

The data presented in this study are available from the corresponding author upon reasonable request.
